# Antibacterial Properties of 3 *H*-Spiro[1-benzofuran-2,1’-cyclohexane] Derivatives from *Heliotropium filifolium*

**DOI:** 10.3390/molecules13102385

**Published:** 2008-10-01

**Authors:** Alejandro Urzúa, Javier Echeverría, Marcos C. Rezende, Marcela Wilkens

**Affiliations:** Facultad de Química y Biología, Casilla 40, Correo 33, Universidad de Santiago de Chile, Santiago, Chile

**Keywords:** 3*H-*spiro[1-benzofuran-2,1’-cyclohexane] derivatives, *Heliotropium filifolium*, antibacterial properties

## Abstract

A re-examination of cuticular components of *Heliotropium filifolium* allowed the isolation of four new compounds: 3’-hydroxy-2’,2’,6’-trimethyl-3*H*-spiro[1-benzo-furan-2,1’-cyclohexane]-5-carboxylic acid (**2**), methyl 3’-acetyloxy-2’,2’,6’-trimethyl-3*H*-spiro[1-benzofuran-2,1’-cyclohexane]-5-carboxylate (**3**), methyl 3’-isopentanoyloxy-2’,2’,6’-trimethyl-3*H*-spiro[1-benzofuran-2,1’-cyclohexane]-5-carboxylate (**4**) and methyl 3’-benzoyloxy-2’,2’,6’-trimethyl-3*H*-spiro[1-benzofuran-2,1’-cyclohexane]-5-carboxylate (**5**). Compounds **2-5** were identified by their spectroscopic analogies with filifolinol (**1**), and their structures confirmed by chemical correlation with **1**. The antimicrobial properties of the compounds were tested against Gram positive and Gram negative bacteria. Some of them proved to be active against Gram positive, but inactive against Gram negative bacteria. In searching for structure-activity relationships from the obtained MIC values, lipophilicity was shown to be an important variable.

## Introduction

Species of the genus *Heliotropium* (Boraginaceae, *Cochranea*) are well represented in Northern and Central Chile. Like many of the plant species of that particular geographic area, they show exudates biosynthesised in specialised glands (trichomes) populating the surface of all of the plants' aerial structures [1]. In addition, other specialised structures produce the waxy coating and additional secretory structures may also be involved

In earlier communications, the isolation of two 3*H*-spiro-1-benzofuran-2,1’-cyclohexanes from the cuticle of *Heliotropium filifolium*: methyl 3’-hydroxy-2’,2’,6’-trimethyl-3*H*-spiro[1-benzofuran-2,1’- cyclohexane]-5-carboxylate (filifolinol, **1**) [2] and the corresponding senecionate ester [3] was reported.

**Figure 1:**
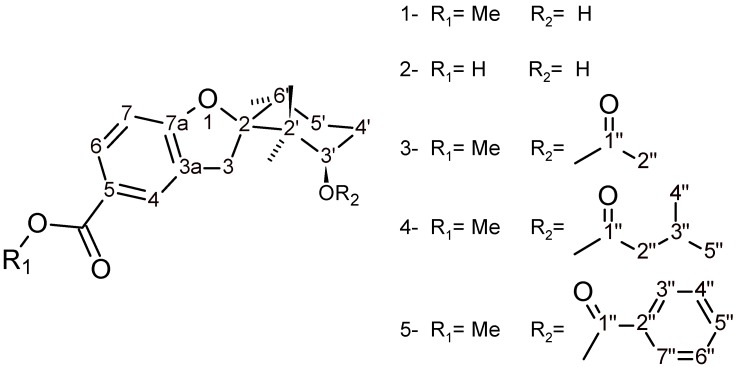
Structures of compounds **1-5**.

A re-examination of cuticular components of the plant has now allowed the isolation of four new compounds: 3’-hydroxy-2’,2’,6’-trimethyl-3*H*-spiro[1-benzofuran-2,1’-cyclohexane]-5-carboxylic acid (**2**), methyl 3’-acetyloxy-2’,2’,6’-trimethyl-3*H*-spiro[1-benzofuran-2,1’-cyclohexane]-5-carboxylate (**3**), methyl 3’-isopentanoyloxy-2’,2’,6’-trimethyl-3*H*-spiro[1-benzofuran-2,1’- cyclohexane]-5-carboxylate (**4**) and methyl 3’-benzoyloxy-2’,2’,6’-trimethyl-3*H*-spiro[1-benzofuran- 2,1’-cyclo-hexane]-5-carboxylate (**5**) (Figure 1). Compounds **2**-**5** were identified by their spectroscopic analogies with filifolinol, and their structures confirmed by derivatizations of **1**.

The antimicrobial properties of the compounds were tested against Gram positive and Gram negative bacteria. They all proved to be inactive against the latter, but some exhibited an interesting activity against Gram positive microorganisms.

## Results and Discussion

Compound **2** was obtained as an amorphous levorotatory solid; the molecular formula C_17_H_22_O_4_ was deduced from its exact mass [M^+^] 290.1518. Its ^1^H- and ^13^C-NMR spectra were similar to those of filifolinol (**1**), but the C-5-carbomethoxy methyl group resonance was absent in both spectra (see Table 1 and Table 2). Extensive 2D NMR experiments (^1^H-^1^H COSY, HMQC, HMBC, NOESY and ^1^H-^1^H COSYLr) supported the structure and permitted complete assignments of all ^1^H- and ^13^C-NMR resonances. In particular the relative stereochemistry of C-3’, C-6’ and C-2 was obtained through NOESY experiments. Irradiation of C-6’-CH_3_, showed correlation with the methylene proton at C-H-3 (3.04 ppm). Irradiation of the C-2’-CH_3 ax_ (1.10 ppm) showed correlation with C-H-3’_ec_ (3.69 ppm). Finally, irradiation of C-H-4’_ax_ showed a strong correlation with C-H-3’ (3.69 ppm). Confirmation of the structure was accomplished by methylation of compound **2** with ethereal CH_2_N_2_ to yield a white solid, m.p: 101-103°; [α]^20^_D_ = -22.7°, identified (TLC, FTIR) as filifolinol (**1**).

**Table 1:** ^1^H-NMR data of methyl 3’-hydroxy-2’,2’,6’-trimethyl-3*H*-spiro[1-benzofuran- 2,1’-cyclohexane]- 5-carboxylate derivatives **2-5**; coupling constants (*J*) in Hz.

Compounds					
H	1 ^a^	2	3	4	5
3	3.04 (1H, d, *J*=17.1)	3.06 (1H, d, *J*=17.6)	3.08 (1H, d, *J*=17.3)	3.07 (1H, d, *J*=17.3)	3.24 (1H, d, *J*=16.8)
	3.62 (1H, d, *J*=17.1)	3.64 (1H, d, *J*=17.6)	3.49 (1H, d, *J*=17.3)	3.50 (1H, d, *J*=17.3)	3.66 (1H, d, *J*=16.8)
4	7.79 (1H, d, *J*=1.2Hz)	7.85 (1H, br s )	7.82 (1H, m)	7.84 (1H, br s)	7.85 (1H, br s)
6	7.83 (1H, dd, *J*_o_=8.1, *J*_m_=1.2)	7.87 (1H,br d, *J*= 9.6)	7.82 (1H, m)	7.83 (1H, br d, J= 7.2)	7.84 (1H, dd, J=6.8; J=3.2)
7	6.71 (1H, d, *J*=8.1)	6.73 (1H, d, *J*=8.8)	6.71 (1H, d, *J*=8.4)	6.72 (1H, d, *J*=8.8)	6.74 (1H, d, *J*=8.4)
3’	3.69 (1H, m)	3.71 ( 1H, br, s)	4.85 (1H, br t, *J*= 2.8)	4.86 (1H, br t, *J*= 2.8)	5.18 (1H br t, *J*= 3.2)
4’	1.62 (1H, m)	1.62 (1H, m)	1.66 (1H, m)	1.49 (1H, m)	1.57 (1H, m)
	1.96 (1H, m)	1.98 (1H, m)	1.92 (1H, m)	1.92 (1H, m)	1.98 (1H, m)
5’	1.52 (1H, m)	1.46 (1H, m)	1.46 (1H, m)	1.49 (1H, m)	1.57 (1H, m)
	1.60 (1H, m)	1.52 (1H, m)	1.52 (1H, m)	1.65 (1H, m)	1.62 (1H, m)
6’	2.28 (1H, m)	2.30 (1H, m)	2.31 (1H, m)	2.30 (1H, m)	2.21 (1H, m)
C-2’-Me(ax)	1.10 (3H, s)	1.02 (3H, s)	0.91 (3H, s)	0.90 (3H, s)	0.99 (3H, s, )
C-2’-Me(eq)	1.14 (3H, s)	1.15 (3H, s)	1.22 (3H, s)	1.23 (3H, s)	1.31 (3H, s)
C-6’-Me	0.78 (3H, d, *J*=7.0)	0.80 (3H, d, *J*=6.8)	0.79 (3H, d, *J*=6.6)	0.79 (3H, d, *J*=6.4)	0.84 (3H, d, *J*=6.8)
OMe	3.89 (3H, s)	-	3.86 (3H, s)	3.87 (3H, s)	3.87 (3H, s)
2’’	-	-	2.11 (3H, s)	2.24 (2H, m)	-
3’’	-	-	-	2.15 (1H, m)	8.06 (1H, m)
4’’	-	-	-	1.00 (3H, dd, *J*_1_=6.4, *J*_2_=2.0)	7.58 (1H, m)
5’’	-	-	-	1.00 (3H, dd, *J*_1_=6.4, *J*_2_=2.0)	7.58 (1H, m)
6’’	-	-	-	-	7.58 (1H, m)
7’’	-	-	-	-	8.06 (1H, m)

^a^ From references [2,3]; all proton resonances were assigned on the basis of COSY, DEPT, HMBC, HSQC and NOESY experiments.

Compound **3** was obtained as a dextrorotatory oil; the molecular formula C_20_H_26_O_5_ was deduced from its exact mass [M^+^] 346.1780. The analysis of the NMR spectra (Table 1 and Table 2) indicated that **3** was the acetylated derivative of filifolinol (**1**). Confirmation of the structure and the stereochemistry of **3** was done by acetylation of filifolinol (**1**), with *N,N*-dimethylaminopyridine and acetyl chloride to yield a pure oil, [α]^20^_D_ = + 0.5°.

**Table 2:** ^13^C-NMR data of, methyl 3’-hydroxy-2’,2’,6’-trimethyl-3*H*-spiro[1-benzofuran- 2,1’-cyclohexane]- 5-carboxylate derivatives **2-5**.

Compounds					
Carbon atom	1 ^a^	2	3	4	5
2	96.92	97.39	96.60	96.62	96.64
3	31.00	31.27	30.95	30.95	31.32
4	126.11	127.06	126.40	126.41	126.44
5	121.52	120.98	122.16	122.15	122.20
6	130.67	131.86	131.08	131.09	131.86
7	107.56	108.04	108.01	108.01	108.07
7a	164.03	164.97	164.15	164.33	164.97
3a	129.09	131.86	128.71	128.71	130.68
2’	42.55	42.89	42.03	42.01	42.40
3’	77.10	77.51	79.18	78.95	79.76
4’	28.85	29.16	26.53	26.56	26.72
5’	25.91	26,18	26.06	26.12	26.32
6’	35.85	36.17	36.06	36.06	36.22
C-2’-Me(ax)	20.48	20,73	20.56	20.63	20.68
C-2’-Me(eq)	22.36	22,64	22.44	22.46	22.69
C-6’-Me	14.90	15.19	15.09	15.08	15.15
C-5-C=O	167.24	171.64	167.31	167.18	167.31
OMe	51.66	-	51.93	51.93	51.96
C=O	-	-	170.28	172.38	166.07
2’’	-	-	21.47	44.10	131.17
3’’	-	-	-	25.49	129.64
4’’	-	-	-	22.45	128.85
5’’	-	-	-	22.49	133.33
6’’	-	-	-	-	128.85
7’’	-	-	-	-	129.64

^a^ From references [2,3]; All carbon resonances were assigned on basis of DEPT, HMBC, HSQC and NOESY experiments.

Compound **4** was obtained as a dextrorotatory oil; the molecular formula C_23_H_32_O_5_ was deduced from its exact mass [M^+^] 388.2250. The analysis of the NMR spectra (Table 1) suggested a derivative of filifolinol (**1**), esterified with a five-carbon acid, identified as isopentanoic acid. Confirmation of the structure and the stereochemistry of **4** was done by esterification of filifolinol (**1**) with *N,N-*dimethyl-aminopyridine and isopentanoyl chloride to yield a pure oil, [α]^20^_D_ = + 12.0°.

Compound **5** was obtained as a dextrorotatory oil; the molecular formula C_25_H_28_O_5_ was deduced from its exact mass [M^+^] 408.1938. The analysis of the NMR spectra (Table 1), suggested a derivative of filifolinol (**1**), esterified with a seven-carbon acid, identified as benzoic acid. Confirmation of the structure and the stereochemistry of **5** was done by benzoylation of filifolinol (**1**) with *N,N*-dimethyl-aminopyridine and benzoyl chloride to yield a pure oil, [α]^20^_D_ = + 92.0°.

Compounds **1**-**5** were evaluated as antimicrobial agents against Gram positive and Gram negative bacteria. They all proved to be inactive against the latter, but some exhibited an interesting activity against Gram positive microorganisms (*Bacillus* *subtilis*, *Bacillus cereus*; *Micrococcus luteus* and *Staphylococcus aureus*), comparable to that of commercial antibiotics like chloramphenicol, ampicillin or tetracycline. Table 3 lists their MIC values against these microorganisms.

**Table 3:** Antibacterial activity of compounds isolated from *H. filifolium*.

Tested Compound	XlogP ^a^	MIC values in liquid media (µg/ml) and solid media (μg)^b^ various microorganisms^c^							
		A		B		C		D	
		µg/mL	µg	µg/mL	µg	µg/mL	µg	µg/mL	µg
**1**	3.29	512	2.5	512	2.5	512	2.5	1024	2.5
**2**	2.97	i	i	i	i	i	i	i	i
**3**	4.03	256	1.25	128	1.25	128	1.25	256	1.25
**4**	5.36	128	0.63	32	0.16	16	0.08	64	1.25
**5**	5.75	16	0.16	32	0.16	16	0.08	16	0.16
Methanol (blank)		i	i	i	i	i	i	i	i
Chloramphenicol		8	1.25	4	2.5	4	1.25	4	1.25
Ampicillin		>1024	i	>1024	i	8	0.16	4	0.08
Tetracycline		128	2.5	32	2.5	8	0.16	4	0.04

^a^ Estimated lipophilicity value [4,5]; ^b^ µg deposited in 5 µL; ^c^ tested microorganisms: A, *Bacillus subtilis* (ATCC 6633); B, *Bacillus cereus* (NAS 569); C, *Micrococcus luteus* (ATCC 9341); D, *Staphylococcus aureus* (ATCC 5638); i, inactive.

In searching for structure-activity trends from the MIC values of Table 3, we investigated the possibility that lipophilicity might be an important factor in the activity of these filifolinol derivatives. This was suggested by the observation that compound **2**, the least active member of the series, possessed two hydrophilic groups, a carboxylic and a hydroxyl function. Conversion of either group, or both, into more hydrophobic ester functionalities led in all cases to more active compounds. Our hypothesis was born out by comparison of the activities of compounds **1**-**5** with their estimated lipophilicities (Table 3). The only inactive compound against all four tested microorganisms was acid **2**, with the smallest lipophilicity, as estimated by its Xlog P value [4]. All other derivatives have higher Xlog P values, and their activity increases in the order **1**<**3**<**4**< **5**, which is the same order of increasing lipophilicities. The good qualitative correlation between lipophilicity and antimicrobial activity does not rule out the importance of other structural factors which may be responsible for the activity of the studied compounds.

## Experimental

### General

Optical rotations were measured on a Perkin-Elmer Polarimeter 241. IR spectra were recorded as KBr discs on a Bruker 66v FT-IR spectrometer. EIMS, HREIMS spectra (direct inlet, EI at 70 eV) were recorded with a FISONS VG AUTOSPEC spectrometer. NMR spectra (both 1D and 2D) were obtained on a Bruker AVANCE 400 spectrometer (400 MHz for ^1^H and 100 MHz for ^13^C, respectively) using the residual solvent peaks as internal standard. Column chromatography was carried out on silica gel (Merck, Kieselgel 60, 60-230 mesh). TLC were carried out on silica gel 60 F254 pre-coated plates with detection accomplished by spraying with p-anisaldehy de followed by heating at 105 °C for 1-2 min.

### Plant material

*Heliotropium filifolium* (Miers) Reiche was collected during the flowering season in October 2006, in Carrizal Bajo, Chile (III Region 28° 45' S, 70° 49' W) and identified by Dr. Sebastián Teiller. Voucher specimens (ST-2214 SSCU) were deposited in the Herbarium of the Faculty of Biological Sciences of Catholic University of Chile, Santiago, Chile.

### Extraction and isolation

The resinous exudate of *H. filifolium* was obtained by dipping the fresh plant material (1 kg) in CH_2_Cl_2_ for 30 s. The extract was concentrated to afford 200 g of a residue. A portion (10 g), was fractionated on silica gel CC eluted with pentane, pentane-CH_2_Cl_2_ (90:10→10:90), CH_2_Cl_2_ and MeOH-CH_2_Cl_2_ (5:95→10:90), yielding eight fractions labelled “F1” to “F8”. Fraction F1 was purified by silica gel CC using pentane-CH_2_Cl_2_ (90:10) to afford methyl-3’-benzoyloxy-2’,2’,6’-trimethyl-3*H*-spiro[1-benzofuran-2,1’-cyclohexane]-5-carboxylate (**5**, 167 mg). Fraction F2 was purified by silica gel CC using pentane-CH_2_Cl_2_ (85:15) to afford methyl-3’-isopentanoyloxy-2’,2’,6’-trimethyl-3 *H*-spiro[1-benzofuran-2,1’-cyclohexane]-5-carboxylate (**4**, 114 mg). Fraction F4 was purified by silica gel CC using pentane-CH_2_Cl_2_ (85:15) to afford methyl-3’-acetyloxy-2’,2’,6’-trimethyl-3*H*-spiro[1- benzofuran-2,1’-cyclohexane]-5-carboxylate (**3**, 257 mg). Fraction F8 was purified by silica gel CC using MeOH-CH_2_Cl_2_ (5:95) to afford 3’-hydroxy-2’,2’,6’-trimethyl-3*H*-spiro[1-benzofuran-2,1’- cyclohexane]-5-carboxylic acid (**2**, 257 mg). Finally, fractions F5, F6 and F7 were crystallized from AcOEt to afford filifolinol (**1**, 3.25 g).

*Methyl 3’-hydroxy-2’,2’,6’-trimethyl-3H-spiro[1-benzofuran-2,1’-cyclohexane]-5-carboxylate* (**1**). Solid, mp 103-105° (EtOAc); [α]^20^_D_ = -22.7 ° (c: 2.1, CHCl_3_), [lit. [2]: mp 102-103° (EtOAc); [α]^22^_D_ = -22.1 ° (c: 0.38, CHCl_3_)]; IR ν_max_ (KBr): 3551 (OH), 1720 (C=O); HREIMS *m/z*: 304.1673 (M^+^, C_18_H_24_O_4_, calcd. 304.1675). The compound was identified by direct comparison with an authentic sample.

*3’-Hydroxy-2’,2’,6’-trimethyl-3H-spiro[1-benzofuran-2,1’-cyclohexane]-5-carboxylic acid* (**2**). Amorphous solid, [α]^20^_D_ = -23.7 ° (c: 2.1, CHCl_3_); IR ν_max_ (KBr): 3358 (OH), 3000-2600, 1675 (COOH), 1607.7 (C=C) cm^-1^; ^1^H-NMR (CDCl_3_) δ: see Table 1; ^13^C-NMR (CDCl_3_) δ: see Table 2; HREIMS *m/z*: 290.1520 (M^+^, C_17_H_22_O_4_, calcd. 290.1518); EIMS m/z (rel. int.): 290 (M ^+^,79%), 273(M^+^_-_ HO, 19%), 245(M ^+^_-_ HO_-_ C=O, 39%), 203 (29), 57 (100).

*Methyl 3’-acetyloxy-2’, 2’, 6’-trimethyl-3 H-spiro[1-benzofuran-2, 1’-cyclohexane]-5-carboxylate* (**3**). Oil, [α]^20^_D_ = + 0.5 ° (c =2.0, CHCl_3_); IR ν_max_ (KBr): 1732.8 (COOR), 1711.7 (COOR), 1608.7 (C=C) cm^-1^; ^1^H-NMR (CDCl_3_) δ: see Table 1; ^13^C-NMR (CDCl_3_) δ: see Table 2; HREIMS *m/z*: 346.1778 (M^+^, C_20_H_26_O_5_, calcd. 346.1780); EIMS m/z (rel. int.): 346 (M ^+^, 5%), 286 (M ^+^_-_ C_2_H_4_O_2_, 100%).

*Methyl 3’-isopentanoyloxy-2’,2’,6’-trimethyl-3H-spiro[1-benzofuran-2,1’-cyclohexane]-5-carboxylate* (**4**). Oil, [α]^20^_D_ = + 12.0 ° (c =2.0, CHCl_3_); IR ν_max_ (KBr): 1733.4 (COOR), 1712.5 (COOR), 1609.9 (C=C) cm^-1^; ^1^H-NMR (CDCl_3_) δ: see Table 1; ^13^C-NMR (CDCl_3_) δ: see Table 2; HREIMS *m/z*: 388.2249 (M^+^, C_23_H_32_O_5_, calcd. 388.2250); EIMS m/z (rel. int.): 388 (M ^+^, 3%), 286 (M ^+^_-_ C_5_H_10_O_2_, 100%).

*Methyl 3’-benzoyloxy-2’,2’,6’-trimethyl-3H-spiro[1-benzofuran-2,1’-cyclohexane]-5-carboxylate* (**5**). Oil, [α]^20^_D_ = + 92.0 ° (c =2.09, CHCl_3_); IR ν_max_ (KBr): 1713.4 (COOR), 1607.7 (C=C) cm^-1^; ^1^H-NMR (CDCl_3_) δ: see Table 1; ^13^C-NMR (CDCl_3_) δ: see Table 2; HREIMS *m/z*: 408.1940 (M^+^, C_25_H_28_O_5_, calcd. 408.1938); EIMS m/z (rel. int.): 408 (M ^+^, 3 %), 286 (M ^+^_-_ C_7_H_6_O_2_, 100%).

Compound **2** (50 mg), was methylated with ethereal CH_2_N_2_ during 24 h at room temperature. Elimination of the ether yield 48 mg of a white solid mp: 101-103°, identified (TLC, FTIR) as filifolinol (**1**). Confirmation of the structures and stereochemistry of **3, 4** and **5** was done by acylation of filifolinol (**1**, 1 mmol) with *N,N*-dimethylaminopyridine (3 mmol) and the appropriate acyl chloride (1.2 mmol) in CH_2_Cl_2_ (50 mL) during 65 h at 0° C. Workup of the reaction mixtures yielded the pure compounds **3**, **4** and **5** (TLC, FTIR, NMR).

### Antibacterial activity determination in solid medium

The antibacterial activity was evaluated against *Bacillus cereus* (NAS 569), *Bacillus subtilis* (ATCC 6633), *Staphylococcus aureus* (ATCC 6538p), *Micrococcus luteus* (ATCC 9341), *Clavibacter michiganensis* subsp. *michiganensis* (Cmm 623), *Escherichia coli* (ATCC 25922), *Salmonella paratyphi* B (ATTCC 2659), *Proteus vulgaris* (ATCC 6380), *Klebsiella pneumoniae* (ATCC 13883) and *Erwinia carotovora* (IC 2610).

The antibacterial activity was determined by the agar overlay method [6]. Bacteria grown overnight in LB-broth [7], were diluted to McFarland 0.5-1.0 (1.5 - 3 x 10^8^ cells/mL) and a portion of this diluate (100 μL) were mixed with molten soft agar (0.7 %, 3 mL) at 50 °C. The soft agar was poured over Petri dishes containing 1.5 % agar (20 mL). Two-fold dilutions of the test samples (5 μL) in methanol were deposited over solidified agar, starting at 1000 μg/mL up to 2 μg/mL. After 18 h of incubation at 37 °C, the diameter of the inhibition zone was determined. Control measurements were carried out with methanol. The minimum inhibitory concentration (MIC) corresponded to the minimum concentration that showed a transparent halo of growth inhibition. The MIC determination was carried out in five independent experiments.

### Antibacterial activity determination in liquid medium

Antibacterial determination in liquid media was carried out by serial two-fold dilutions of the compounds **1**-**5** and the antibiotics tetracycline, chloramphenicol and ampicillin in 2 mL in a range of 1024 µg/mL to 4 µg/mL. Twenty five microliters of bacteria culture (McFarland 1.0 of *B. cereus*, *B. subtilis*, *S. aureus* and *M. luteus*) was added to each compound concentration, incubated at 37° C for 18 h and the minimal inhibitory concentration was registered. The MIC determination was carried out in five independent experiments.

### Estimated lipophilicity values

Lipophilicity values have been estimated with the aid of the XLOGP3 program, available in the web [4]. The XLOGP3 method estimates log P values for structures related with reference compounds for which experimental log P values are available [5]. The additive model implemented in XLOGP3 uses a total of 87 atom/group types and two correction factors as descriptors. It is calibrated on a training set of 8199 organic compounds with reliable log*P* data through a multivariate linear regression analysis. It compares favourably with other methods, with average errors in the range of ± 0.24-0.51 units.
